# Identification and Characterization of a Candidate *Wolbachia pipientis* Type IV Effector That Interacts with the Actin Cytoskeleton

**DOI:** 10.1128/mBio.00622-16

**Published:** 2016-07-05

**Authors:** Kathy B. Sheehan, MaryAnn Martin, Cammie F. Lesser, Ralph R. Isberg, Irene L. G. Newton

**Affiliations:** a Department of Biology, Indiana University, Bloomington, Indiana, USA; b Department of Medicine, Division of Infectious Diseases, Massachusetts General Hospital, Harvard Medical School, Cambridge, Massachusetts, USA; c Department of Molecular Biology and Microbiology, Tufts University School of Medicine, Boston, Massachusetts, USA

## Abstract

Many bacteria live as intracellular symbionts, causing persistent infections within insects. One extraordinarily common infection is that of *Wolbachia pipientis*, which infects 40% of insect species and induces reproductive effects. The bacteria are passed from generation to generation both vertically (through the oocyte) and horizontally (by environmental transmission). Maintenance of the infection within *Drosophila melanogaster* is sensitive to the regulation of actin, as *Wolbachia* inefficiently colonizes strains hemizygous for the profilin or villin genes. Therefore, we hypothesized that *Wolbachia* must depend on the host actin cytoskeleton. In this study, we identify and characterize a *Wolbachia* protein (WD0830) that is predicted to be secreted by the bacterial parasite. Expression of WD0830 in a model eukaryote (the yeast *Saccharomyces cerevisiae*) induces a growth defect associated with the appearance of aberrant, filamentous structures which colocalize with rhodamine-phalloidin-stained actin. Purified WD0830 bundles actin *in vitro* and cosediments with actin filaments, suggesting a direct interaction of the two proteins. We characterized the expression of WD0830 throughout *Drosophila* development and found it to be upregulated in third-instar larvae, peaking in early pupation, during the critical formation of adult tissues, including the reproductive system. In transgenic flies, heterologously expressed WD0830 localizes to the developing oocyte. Additionally, overexpression of WD0830 results in increased *Wolbachia* titers in whole flies, in stage 9 and 10 oocytes, and in embryos, compared to controls, suggesting that the protein may facilitate *Wolbachia*’s replication or transmission. Therefore, this candidate secreted effector may play a role in *Wolbachia*’s infection of and persistence within host niches.

## IMPORTANCE

The obligate intracellular *Wolbachia pipientis* is a ubiquitous alphaproteobacterial symbiont of arthropods and nematodes and is related to the rickettsial pathogens *Ehrlichia* spp. and *Anaplasma* spp. Studies of *Wolbachia* cell biology suggest that this bacterium relies on host actin for efficient proliferation and transmission between generations. Here, we identified and characterized a *Wolbachia* protein that localizes to and manipulates the eukaryotic actin cytoskeleton, is expressed by *Wolbachia* during host development, and alters *Wolbachia* titers and localization in transgenic fruit flies. We hypothesize that WD0830 may be utilized by the bacterium to facilitate replication in or invasion of different niches during host development.

## INTRODUCTION

*Wolbachia pipientis* is a ubiquitous alphaproteobacterium that is related to the rickettsial pathogens *Ehrlichia* spp. and *Anaplasma* spp. and that infects arthropods and nematodes (1). *Wolbachia pipientis* causes a persistent infection within insects, often inducing reproductive effects including sperm-egg incompatibility, male killing, and feminization (1). *Wolbachia* spp. have received attention recently due to their medical relevance, as the bacteria protect insect hosts from RNA virus infection and are currently being implemented to prevent transmission of dengue virus from mosquitoes to humans (2, 3). Additionally, drugs that promote *Wolbachia* clearance are being investigated as potential therapeutics for filarial nematode infection (4, 5). For example, the antibiotics tetracycline, rifampin, and doxycycline reduce the ability of these nematodes to reproduce (5).

Intracellular bacteria share a common need to manipulate the host cell for survival. Many accomplish this through the use of secretion systems, nanomachines that enable the microbes to directly transfer proteins from bacterium into the cytosol of host cells. These proteins, referred to as effectors, often act to manipulate or usurp host cell processes in order to promote bacterial infection (6–8). While effectors are bacterial in origin, they act within eukaryotic cells and hence often encode domains that share structural and sometimes sequence similarity with eukaryotic proteins (6, 8–10). Based on analyses of the genome sequences of various *Wolbachia* strains, it is known that *Wolbachia* symbionts likely encode a functional type IV secretion system (11) homologous to the type IV-1-A system of *Agrobacterium* spp. (12). Furthermore, there is evidence that the genes encoding this putative secretion system are expressed by *Wolbachia* within its natural host (13). For example, transcripts for the nine type IV *vir* genes are highly expressed by *Wolbachia* throughout host development (14). The results of this transcriptome analysis supported a model in which the type IV machinery is constitutively expressed, while the candidate secreted effectors are differentially regulated during development (14). Therefore, it is likely that *Wolbachia* harbors effectors that are used to manipulate the host cell. Indeed, *Wolbachia* genomes encode many proteins with eukaryotic domains (15). Identification and characterization of *Wolbachia* effectors will allow us to better understand the basic biology of infection, possibly including how *Wolbachia* induces reproductive effects.

Important to *Wolbachia*’s ability to induce reproductive effects generation after generation is its ability to persist within and be passed through the host germ line. In this regard, there is evidence that *Wolbachia* utilizes the host cytoskeleton, both microtubules (16, 17) and actin (18), to achieve transmission. Additionally, the bacterium undergoes somatic cell-to-germ line transmission, i.e., *Wolbachia* injected into the abdominal cavity of *Drosophila melanogaster* transits to the germ line (19). The ability of *Wolbachia* to pass through layers of host tissues as well as into and out of nonphagocytic cells is hypothesized to involve manipulation of the host actin cytoskeleton (20–22). Consistent with this hypothesis, in nematodes the transit of *Wolbachia* into germ cells is correlated with a weakening of cortical actin (21), and *Wolbachia* transmission in flies is sensitive to the regulation of actin in the host. For example, flies that carry mutations in profilin and villin, two actin regulatory proteins, exhibit low-titer infections and inefficient bacterial transmission to progeny (18, 23).

Here, we characterize WD0830, a well-conserved *Wolbachia* protein that contains an α-synuclein domain, a eukaryotic domain known to interact with actin. α-Synuclein, the mammalian homolog, colocalizes with actin filaments *in vivo* (24), and in quantitative proteomics assays it has been found to interact with components of the cytoskeleton (such as cofilin and destrin) (25). We observed that when ectopically expressed in *Saccharomyces cerevisiae*, WD0830 localizes to and manipulates the yeast actin cytoskeleton, resulting in growth inhibition. In addition, we demonstrate that purified WD0830 binds to and bundles filamentous (F)-actin in cosedimentation assays*.* During a native infection, WD0830 transcripts are upregulated in adults, larvae, and pupae, with the highest expression in early pupal development. Finally, in female transgenic flies harboring *Wolbachia* and overexpressing WD0830, the *Wolbachia* protein localizes to developing oocytes. In these same flies, *Wolbachia* accumulates to higher titers than genetic controls, with *Wolbachia* localizing more strongly to the developing oocyte. This effect spans generations, as offspring from these transgenic lines lay eggs with increased *Wolbachia* titers compared to controls. Based on these observations, we propose that WD0830 functions to manipulate actin during host development and facilitates *Wolbachia* replication and transit to different niches.

## RESULTS

### Identification of a *Wolbachia* protein with a region showing sequence similarity to α-synuclein.

**FIG 1:**
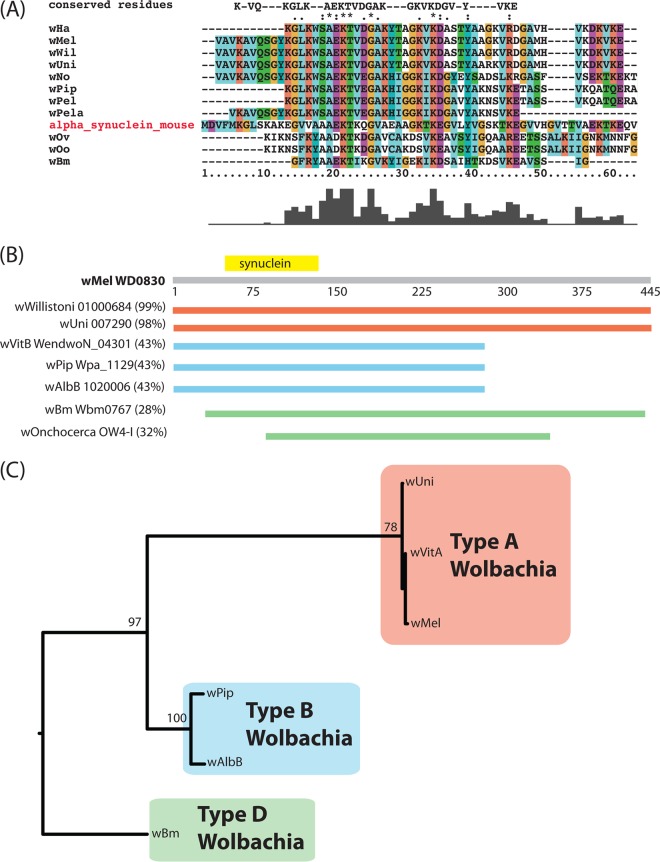
The α-synuclein domain-containing protein is conserved across the *Wolbachia* genus. (A) Alignment of the synuclein domain from WD0830 and *Wolbachia* homologs compared to mammalian alpha-synuclein (mouse). Conserved residues are shown at the top of the alignment. (B) Conservation (the percent protein identity) and length of the WD0830 homolog across the *Wolbachia* genus. Area and percent similarity are indicated by the colored line across the WD0830 open reading frame. (C) Phylogeny of these same open reading frames generated from codon alignments (ClustalW) edited by eye and then used as input to RAxML (GTR+γ) recovered major *Wolbachia* clades.

Bacterial pathogens commonly utilize secretion systems to manipulate their hosts through the secretion of effectors into the host cytosol. Although no such effector has yet been described for an obligate intracellular symbiont, *Wolbachia* genomes encode many proteins containing eukaryotic domains and sequence similarities to eukaryotic proteins. We hypothesize that well-conserved *Wolbachia* proteins that share sequence similarities with eukaryotic domains may be secreted effectors. Through similarity searches using Pfam and NCBI’s GenBank, we identified, in genomes from *Wolbachia* types A, B, C, and D, a conserved protein containing an α-synuclein domain (Fig. 1). This domain is common to proteins found in vertebrates and is known to mediate interactions with actin (24–28). The sequence similarity between the N terminus of WD0830 and mammalian α-synuclein (GenBank accession number AF253513) is 33% identity across the relevant amino acids, and this particular protein is well conserved within the *Wolbachia* clade. This protein contains no other domain homology and the extent of conservation across the *Wolbachia* phylogeny varies, with *w*Uni (from *Muscidifurax uniraptor*), *w*VitA (from *Nasonia vitripenis*), and *w*Mel (from *Drosophila melanogaster*) sharing nearly 97% identity, in contrast to the most divergent homolog, that from type D *Wolbachia*, which shares ~30% (Fig. 1). Conservation within the synuclein domain correlates with the overall percent identity between homologs (Fig. 1). The closest non-*Wolbachia* homolog found (within GenBank’s nr database) is a hypothetical protein from the invasive pathogen *Providencia alcalifaciens* (30% identity, 29% coverage) (29, 30). After *Providencia*, the other best top BLAST hits are all eukaryotic organisms (e.g., *Adienta vaga*, *Plasmodium falciparum*, *Caenorhabditis elegans*, *Dictyostelium discoideum*).

### The *w*Mel protein WD0830 elicits a growth defect in yeast and colocalizes with actin.

**FIG 2:**
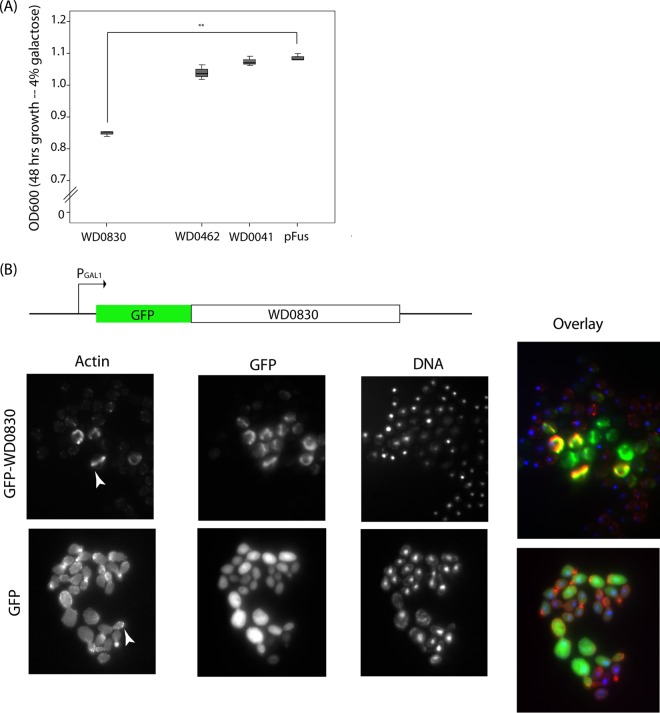
Expression of *Wolbachia* protein WD0830 in yeast. (A) Yeast cells carrying plasmids that conditionally expressed GFP, GFP-WD0830, or two other *Wolbachia* proteins (WD0462 and WD0041) were grown for 48 h under inducing conditions (4% galactose; results are the means of 3 replicate experiments). **, *P* < 0.001 (*t* test, comparing final OD achieved by strains expressing GFP-WD0830 versus GFP alone). (B) Representative images of yeast cells expressing GFP-WD0830 or GFP alone and stained with rhodamine-labeled phalloidin. Arrowheads point to cortical actin punctae in control yeast cells (GFP only) and actin filaments in GFP-WD0830-expressing yeast.

When expressed in the yeast *Saccharomyces cerevisiae*, bacterial effectors, but not housekeeping proteins, often result in growth inhibition due to conserved targeting of eukaryotic cellular processes (31–33). Thus, given the genetic intractability of *Wolbachia* and the lack of any *in vitro* assays to identify secreted proteins, we next investigated how WD0830 behaves when expressed in yeast (see Fig. S1 in the supplemental material)*.* The growth of yeast expressing a green fluorescent protein (GFP)-WD0830 fusion protein was markedly suppressed compared to expression of GFP alone, supporting the potential role of WD0830 as a secreted substrate (Fig. 2A). This statistically significant growth defect (*P* < 0.0001) was not observed in yeast that harbored clones encoding two other *Wolbachia* hypothetical proteins (WD0041 or WD0462) (Fig. 2A).

Given that effectors often exhibit similar subcellular localization patterns when expressed in yeast and mammalian cells (31, 33–35), using fluorescence microscopy we next investigated the subcellular localization pattern of the GFP-WD0830 fusion protein when expressed in yeast. As shown in Fig. 2B, GFP-WD0830 localized to filamentous structures within the yeast cell (Fig. 2B). This localization is reminiscent of actin filaments observed in wild-type yeast expressing the *Salmonella enterica* serovar Typhimurium type III secreted effector SipA, a protein that promotes bundling of actin filaments (31, 36–39). We therefore stained the actin cytoskeleton of yeast that express GFP or GFP-WD0830 with rhodamine-labeled phalloidin. As shown in the GFP-alone panel in Fig. 2B, the yeast actin cytoskeleton normally comprises cortical actin patches and, in polarized cells, actin filaments (which can be difficult to visualize). These structures are no longer observed in yeast that express GFP-WD0830. Rather, we observed thick cables that colocalized with the labeled actin, structures similar to those previously observed with expression of a *Salmonella* type III secreted effector, SipA, in yeast (31).

### WD0830 interacts directly with and bundles F-actin.

**FIG 3:**
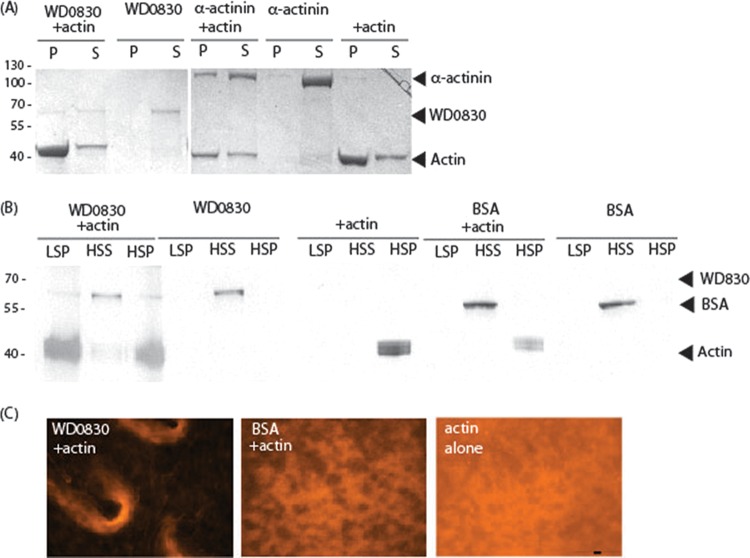
WD0830 cosediments with and bundles actin. (A) Purified WD0830 and alpha-actinin (positive-control actin binding protein) were incubated with polymerized rabbit skeletal actin and subjected to centrifugation at 150,000 × *g*, fractionated by SDS-PAGE, and silver stained to visualize proteins in the supernatant (S) and the pellet (P). (B) To identify actin bundling activity, polymerized rabbit skeletal actin was incubated with or without WD0830 as well as with or without BSA (as a negative control) and subjected to low-speed (14,000 × g) centrifugation before high-speed (150,000 × g) centrifugation. LSP, low-speed pellet; HSS, high-speed supernatant; HSP, high-speed pellet. (C) To visualize actin bundling, polymerized rabbit skeletal actin was incubated with either WD0830 or BSA and then directly stained with Acti-stain 555, mounted on a slide, and visualized by fluorescence microscopy. Bar, 100 µm. All images were taken at the same magnification.

Based on the localization of GFP-WD0830 in yeast, we hypothesized that this *Wolbachia* protein directly binds to F-actin (F for filamentous). To test this hypothesis, we investigated whether WD0830 purified from *Escherichia coli* directly bound purified actin filaments in a sedimentation assay. In this assay, proteins that bind to F-actin will cosediment and thus pellet after ultracentrifugation. We therefore tested and compared the ability of *E. coli* purified WD0830 (see Fig. S2 in the supplemental material) and alpha-actinin, a well-characterized action binding protein (40, 41), to directly interact with polymerized rabbit skeletal muscle actin (Cytoskeleton, Inc.). As a negative control, we also included bovine serum albumin (BSA), as recommended in the standard protocol (42–44). In the cosedimentation assay, proteins were incubated with polymerized actin and, after subjecting the proteins to centrifugation at 150,000 × *g*, both supernatants and pellets were separated by and visualized in a silver-stained SDS-PAGE gel. Proteins that directly interact with actin are found in the pellet fraction only when actin is present. WD0830 and alpha-actinin both cosedimented with actin (P fractions) (Fig. 3A). The amount of *Wolbachia* WD0830 protein detected in the pellet was 24% (±10% [standard deviation]; *n* = 3) of the total when actin was present, compared to 3% (±2%; *n* = 3) without actin present (Fig. 3A), consistent with direct binding. This enrichment is in the same range as observed for alpha-actinin, our positive control (28% in the pellet with actin, compared to 4% in the pellet without actin). We did not observe any sedimentation of BSA with actin (Fig. 3B). This result suggests that WD0830 directly interacts with actin.

Because GFP-WD0830 in yeast appeared to generate actin filaments similar to those generated by the *Salmonella* effector SipA, an actin-bundling protein, we compared the ability of WD0830 to bundle actin, as assessed in a low-speed sedimentation assay (45). Strikingly, only in the presence of WD0830 did F-actin sediment at low speed (14,000 × *g*) (Fig. 3B, LSP), consistent with WD0830 bundling actin. We then visualized the state of actin filaments after incubation with WD0830, with BSA or without additional proteins, and compared the results to incubation alone by using fluorescence microscopy. In the presence of WD0830, but not BSA, we observed F-actin bundles (Fig. 3C).

### Characterization of native and ectopic WD0830 expression during *Drosophila* development.

**FIG 4:**
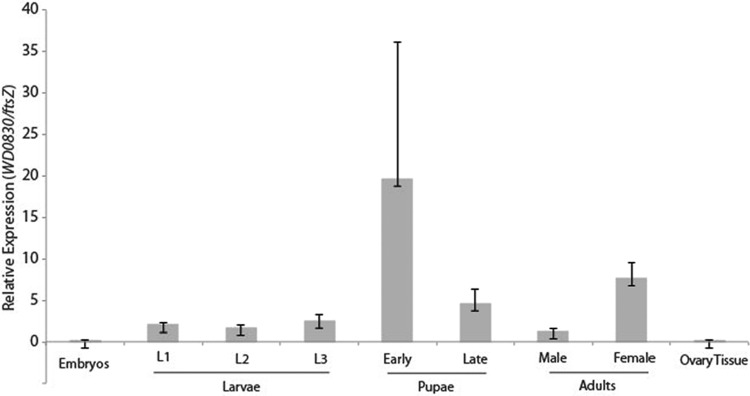
Expression of WD0830 during fly development. Shown are the results of a qRT-PCR analysis of *Wolbachia*-infected flies at noted stages of development (each developmental stage was represented by 5 biological replicates). WD0830 (WalE1) expression is presented relative to that of *Wolbachia* FtsZ at each developmental stage.

Next, to determine the levels of WD0830 expression during a natural infection, we harvested RNA from *Wolbachia*-infected *Drosophila* at seven different time points during fly development: embryos, first to third instar, early and late pupae, and adults (male and female). We quantified WD0830 expression, normalizing levels to those of the *ftsZ* gene by using quantitative reverse transcription-PCR (qRT-PCR). FtsZ is a core conserved bacterial protein involved in cell division and is known to be highly expressed throughout host development (14), making it an appropriate reference for transcription rates relative to bacterial growth. We found that expression of WD0830 relative to that of *ftsZ* was upregulated during pupation, the developmental period during which ovary development begins and larval prepupal ovaries differentiate into the well-characterized adult structures (46), and thus a critical time point during *Drosophila* development (Fig. 4). Components of secretion systems, including the *inv/spa* genes, which encode the type III machinery, have been shown to be upregulated during host pupal development in other facultative intracellular symbionts (47), although the genes encoding the machinery of the *Wolbachia* type IV components have also been observed to be constitutively expressed throughout the host life cycle (14). Our data indicate that WD0830, relative to bacterial cell division, was most highly expressed during pupal development (*P* < 0.05), coincident with the development and maturation of important adult structures, such as the reproductive system.

**FIG 5:**
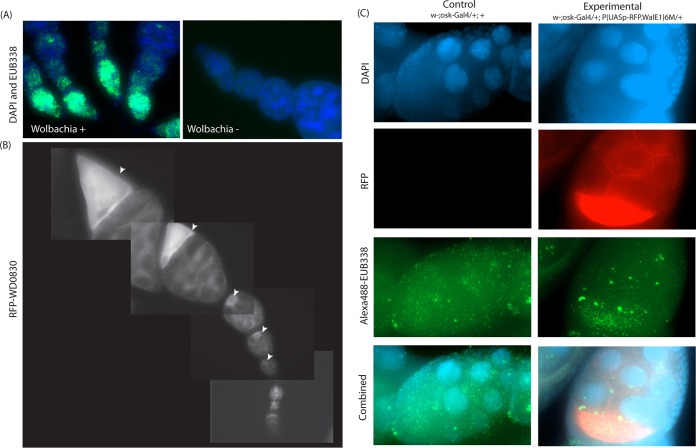
Expression of *Wolbachia* protein WD0830 in transgenic *Drosophila melanogaster* during oogenesis increases *Wolbachia* localization to the developing oocyte*.* (A) Fluorescent *in situ* hybridization probe EUB338 stained *Wolbachia* in infected flies (stock 145) but not uninfected flies (stock 25211). Green signal is from EUB338-Alexa 488, and blue signal is from host nuclei (DAPI stained). (B) A montage of four single-plane fluorescence microscopic images (raw images, unaltered) for visualizing RFP-WD0830 in transgenic flies, expressed using the Osk-Gal4 driver. RFP-WD0830 localized to the developing oocyte and maintained this localization during oogenesis (arrowheads). (C) Localization of *Wolbachia* and RFP-WD0830 in stage 9 to 10 oocytes in egg chambers from control (w−;osk-GAL4/+;+) flies expressing GAL4 alone under control of osk or from experimental (w−;osk-GAL4/+;P{UASp-RFP.WALE1}6 M/+) transgenic flies, in which RFP-WD0830 expression is driven under control of osk-GAL4. These tissues were stained for chromosomal DNA (using DAPI) and *in situ* probed for *Wolbachia* using EUB338.

WD0830 is expressed during a natural infection and during key time points (e.g., in the development of the reproductive organs). Because *Wolbachia* colonizes the reproductive tract and the actin cytoskeleton influences maternal transmission (18), we next investigated if the heterologous expression of WD0830 affected the dynamics of a *Wolbachia* infection. *Drosophila* is an excellent model insect system in which to study *Wolbachia* infection. The primary vertical colonization of flies by the bacterium occurs during oogenesis (19). Development of the oocyte begins in the anterior tip of the ovary, in a region called the germarium, a structure containing the germ line stem cells from which oocytes differentiate (48). *Wolbachia* can be observed throughout progressive stages of oocyte development within a single egg chamber and in the reproductive tissues (Fig. 5A).

**FIG 6:**
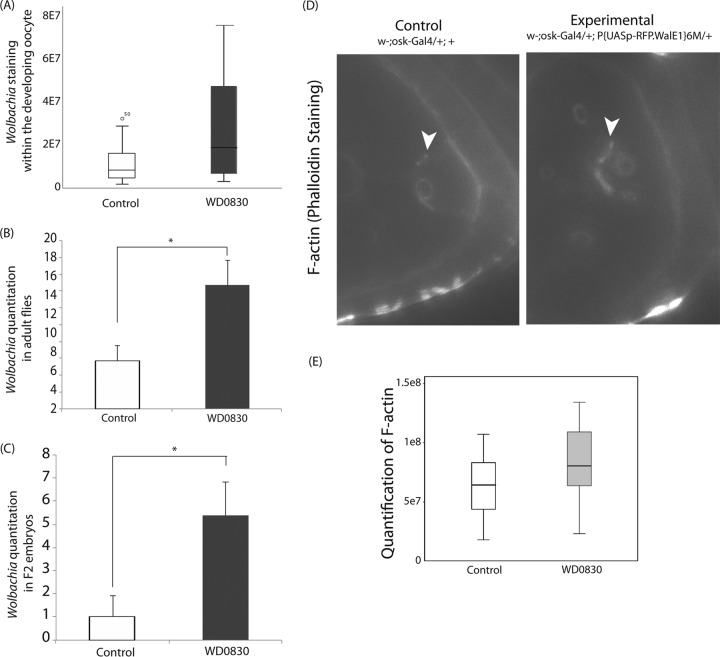
Expression of WD08330 in the reproductive tract increases the density of *Wolbachia* in both transgenic flies and their progeny. (A) The density of *Wolbachia* localizing to the developing oocyte was increased when WD0830 was expressed (as measured by EUB338 staining [see Materials and Methods]) (*n* ≥ 25 for each background; *t* test, *t* = 3.565, df =32.055; *P* = 0.001). (B) *Wolbachia* density in whole transgenic flies was increased (assessed by qPCR for *wsp/Rpl32*) relative to control flies (*t* test, *t* = 2.65, df =6; *P* = 0.038). (C) Six-hour embryos from transgenic flies (F_2_) had greater *Wolbachia* loads (assessed by qPCR) than genetic controls (difference in *wsp*/*Rpl32*, 3.4- to 16.8-fold; *t* test, *t* = 2.530, df =12.362; *P* = 0.026). (D) The amount of F-actin staining (based on fluorescent phalloidin binding) in ring canals adjacent to developing oocytes was altered upon RFP-WD0830 expression. Representative actin ring canals in transgenic flies expressing WD0830 (w−; osk-GAL4/+;P{UASp-RFP.WalE1}6 M/+) compared to genetic controls (w−;osk-GAL4/+;+). (E) Expression of RFP-WD0830 increased the staining associated with actin ring canals adjacent to the developing oocyte (*n* ≥ 24 for each genotype; *t* = 2.8314, df =47; *P* = 0.006).

We overexpressed a red fluorescent protein (RFP)-WD0830 fusion protein in *Wolbachia*-infected transgenic flies by using a variety of drivers (osk-GAL4, MTD-GAL4, matα4-GAL4). For each of these drivers, we observed the same localization of the expressed protein (Fig. 5B; osk-GAL4 and UAS-RFP-WD0830 data are shown as representative). RFP-WD0830 localized to the developing oocyte early and maintained this localization throughout oogenesis (Fig. 5B). Expression of WD0830 in transgenic flies did not result in gross differences in fly fecundity; the number of progeny between osk-GAL4;RFP-WD0830 flies and genetic controls did not significantly differ (*t* test, *t* = 1.486, df =17.076; *P* = 0.155). In addition, we quantified the density of *Wolbachia* in the developing oocyte by using fluorescence *in situ* hybridization (Fig. 5A), and we did not observe a significant difference in *Wolbachia* density in entire stage 9 to 10 egg chambers between control and transgenic flies (*n* ≥ 25 for each background; *P* > 0.05). However, the density of *Wolbachia* found within the developing oocyte was statistically significantly increased in RFP-WD0830-expressing flies compared to genetic controls (*n* ≥ 25 for each background; *t* = 3.565, df =32.055; *P* = 0.001) (Fig. 5C and 6A). This higher-titer infection was also observed by utilizing qPCR on whole transgenic female flies overexpressing WD0830 compared to control flies (*wsp/rpl32*; *t* = 2.65, df =6; *P* = 0.038) (Fig. 6B).

Because the localization of WD0830 correlated with increased *Wolbachia* staining in developing oocytes, we next investigated if embryos derived from transgenic females overexpressing WD0830 harbored higher *Wolbachia* titers. Using qPCR (for *wsp/Rpl32*) on 6-h embryos, we found that when transgenic flies expressed WD0830, their embryos harbored a greater quantity of *Wolbachia* than seen in genetic controls (with an increase between 3.4- and 16.8-fold for comparisons between embryos from three independent, transgenic lines expressing WD0830 and F_1_ embryos from control crosses) (Fig. 6C). Therefore, ectopic expression of WD0830 in an infected *Drosophila melanogaster* germ line increases the *Wolbachia* titer in the presumptive oocyte and may increase the copy numbers of *Wolbachia* detected in the next generation (as we showed via qPCR).

We found that overexpression of WD0830 in yeast corresponded to a change in the organization of the cortical F-actin cytoskeleton. We therefore sought to characterize changes in the F-actin skeleton in transgenic flies. Nurse cells transfer their cytoplasmic contents, through F-actin-derived structures termed ring canals, into the developing oocyte. This process is called cytoplasmic dumping, and *Wolbachia* is thought to be delivered to the oocyte via this same route (16). We investigated potential changes to the amount of F-actin associated with ring canals (based on fluorescent phalloidin staining) when RFP-WD0830 was overexpressed. We observed RFP-WD0830 accumulating in the cytoplasm of the developing oocyte (Fig. 5B), and in early egg chambers (stages 5 to 9), RFP-WD0830 expression resulted in thicker actin ring canals adjacent to the developing oocyte (Fig. 6D). Overall, expression of RFP-WD0830 resulted in a 30% increase in the amount of F-actin staining in ring canals adjacent to the oocyte (*n* ≥ 24 for each genotype; *t* = 2.8314, df =47; *P* = 0.006) (Fig. 6E). Regardless of the stage examined, RFP-WD0830-expressing flies exhibited more fluorescent phalloidin staining in actin ring canals than did the genetic controls (with an observed maximal 2-fold increase in stage 5 and 6 oocytes). However, and importantly, we did not observe enrichment of RFP-WD0830 on these same actin ring canals. Therefore, although this modest increase in F-actin staining was statistically significant in transgenic animals overexpressing RFP-WD0830, it remains to be determined if this difference is biologically relevant and behind the observed phenotype. Also, although RFP-WD0830 bundles actin *in vitro*, its natural function in the developing oocyte has yet to be determined.

## DISCUSSION

Recent cell biological evidence suggests that *Wolbachia* may coopt host actin during infection. The intracellular bacterium is able to enter and exit host cells during the course of development. In both worms and flies, *Wolbachia* undergoes somatic cell-to-germ line transmission (19, 49), and in worms, this transmission has been visually correlated with a reduction in the integrity of cortical actin (21). In flies, *Wolbachia* is sensitive to the regulation of actin, such that heterozygous mutants in key regulatory proteins are unable to efficiently transmit the parasite between generations (18, 23). In order to identify candidate *Wolbachia* proteins that may be responsible for cell invasion, we focused on a *Wolbachia* protein, WD0830, which contains a eukaryotic α-synuclein domain and which we named *Wolbachia* actin-localizing effector 1 (WalE1).

WalE1 is conserved across a diverse range of *Wolbachia* strains, is specific and unique to the genus, and contains a region at the N terminus that has sequence similarity to synuclein (Fig. 1). *Wolbachia* strains from four major supergroups encode WalE1 homologs: clades A (*w*Uni, *w*Will), B (*w*Pip, *w*AlbB, *w*VitB), C (*w*Onch), and D (*w*Bm). Heterologously expressed WalE1 colocalizes with actin in yeast and induces a growth defect, consistent with the properties of other known bacterium-secreted effectors. WalE1 binds to and bundles actin *in vitro*, pointing to a direct interaction with the host cytoskeleton. During a natural infection, *Wolbachia* expresses WalE1 transcripts at critical stages during fly development, and when overexpressed in transgenic flies, WalE1 localizes to the developing oocyte and increases *Wolbachia* titers in developing oocytes and embryos derived from these flies (Fig. 4 and 5). In addition, WalE1 is translocated through a surrogate type IV secretion system (P. J. Christie and I. L. G. Newton, unpublished data). Combined, our evidence suggests that *Wolbachia* may utilize WalE1 during development to manipulate host actin and facilitate replication in and infection of important niches, such as the reproductive tissues and the developing oocyte.

### Sequence similarities between WalE1 and other bacterial effectors.

The manipulation of actin by invading, intracellular bacteria is not uncommon; in order for these bacteria to travel from one cell to another, they must possess the ability to manipulate actin. Many important human pathogens either recruit host actin binding proteins or directly interact with actin (50–53). For example, toxins that covalently modify actin are produced by a variety of Gram-positive (54–56) and Gram-negative bacteria (57). Other strategies for altering the host cytoskeleton include indirect methods that either recruit host actin nucleating proteins (such as Arp2/3) (58–60), alter host actin binding proteins (such as fodrin) (61), or act on the cytoskeleton through other pathways (such as G-protein signaling cascades) (62, 63). Bacterial proteins that directly affect actin polymerization include the WH2 domain-containing actin nucleators (TARP proteins in *Chlamydia* [64] and the VopL effector in *Vibrio parahaemolyticus* [65–67]), the VipA effector in *Legionella*, which also affects organelle trafficking (68), and the SipC/SspC homologs found within some enteric bacteria (such as *Shigella* spp. [69]) and originally identified and characterized in *Salmonella enterica* serovar Typhimurium (70). The *Salmonella* protein SipC nucleates filament formation and increases the rate of F-actin formation (70), while another *Salmonella*-secreted effector, SipA, decreases the critical concentration of actin and inhibits the depolymerization of filaments (71). Importantly, WalE1 does not contain any region homologous to either WH2, the so-called VopL C-terminal domain (VCD), *Legionella*’s VipA effector, or *Salmonella*’s SipA or SipC. Additionally, WalE1 does not contain any of the other known domains involved in actin nucleation by bacteria (e.g., FH2) (72). Therefore, WalE1 may represent a novel evolved strategy for bacterium-host interactions.

### Model for WalE1 function during development of the host.

During host development, *Wolbachia* has been observed to segregate between host cells during mitotic divisions and to migrate between different tissues and niches in order to achieve the localization observed in adult reproductive tissues (21, 49). *Wolbachia* soma-to-germline transmission has been observed in many different systems, including *in vivo* injection of *Wolbachia* into *Drosophila* (19) and *in vitro* infection of *Anopheles gambiae* egg chambers (73). In order to achieve this cell-to-cell transmission, *Wolbachia* likely manipulates host actin. WalE1 is the first *Wolbachia* protein identified to bind to and modify actin *in vitro* and alter infection dynamics *in vivo. walE1* expression is upregulated during critical stages of host development, and WalE1-transgenic flies produce oocytes and embryos with larger quantities of *Wolbachia*. Based on this evidence, we propose a model in which WalE1 is used by *Wolbachia* to manipulate host actin directly. Further work will identify other host targets of WalE1 and characterize the biochemistry of WalE1’s interaction with eukaryotic actin.

### Summary.

Evidence presented here suggests that *Wolbachia* encodes a candidate secreted effector which interacts with and manipulates eukaryotic actin. WalE1 is the first putative *Wolbachia* effector characterized and is the only actin-manipulating protein identified from a nonpathogenic bacterium. Our results suggest new avenues of research in *Wolbachia* cell biology for the investigation of actin as a host cytoskeletal element of importance in the context of symbiosis.

## MATERIALS AND METHODS

### Bioinformatics and evolutionary analyses.

The annotated *Wolbachia w*Mel genome (AE017196) was the source of the query sequence of WD0830 used in reciprocal BLAST experiments. Orthologous WD0830 sequences between type A, B, and D *Wolbachia* strains were generated using reciprocal BLAST against the following genomes (*w*VitA, PRJNA213627; *w*Uni, BioProject accession number PRJNA33275; WBM, NC_006833.1; *w*Pip-Pel, NC_010981.1; *w*AlbB, CAGB00000000.1). Protein-coding regions of orthologs were then aligned using ClustalW (74), and maximum-likelihood trees were generated using RAxML (75) within PROTGAMMA (BLOSUM62 matrix), generating 1,000 bootstrap replicates.

### Amplification, cloning, and transformation of *w*Mel genes.

Genes from the *w*Mel genome were amplified using modified forward primers to facilitate cloning with the Gateway pENTR-D/TOPO system (Invitrogen), following the manufacturer’s instructions, and transformed into One Shot Top10 competent cells (Invitrogen) using standard protocols. Transformations were plated on selective plates, and entry vector constructs generated by this reaction were sequence verified to confirm that protein products generated were in frame and correctly cloned. Correct entry vectors were used in combination with the *Pfu* yeast destination vector (76) in an LR clonase (Invitrogen) reaction as described in the user manual, and these resultant expression vectors were verified by restriction enzyme digests and sequencing.

### Yeast molecular biology, quantitative growth assays, and microscopy.

Yeast strain S288C (BY4741; *MAT***a**) was transformed with sequence-verified expression vectors generated using the polyethylene glycol-lithium acetate method (77). Yeast transformants were inoculated into selective synthetic medium with 2% (wt/vol) glucose. These cultures were grown overnight to saturation (at 30°C) before transfer into medium containing 2% raffinose. After cultures reached an optical density at 600 nm (OD_600_) of 0.3 to 0.4, they were pinned into selective synthetic medium containing 2% galactose (to induce expression) or 2% glucose (to repress expression). These growth assays were performed in triplicate. Optical densities of yeast growing under each condition were measured using an Epoch plate reader (Biotek Instruments, VT) after 24, 36, and 48 h of growth at 30°C.

Yeast harboring the expression vectors containing *Wolbachia* GFP fusions were grown overnight in selective synthetic medium containing 2% raffinose. Optical density measurements were taken, and the yeast were diluted to an OD_600_ of 0.1 in synthetic medium containing 2% galactose to induce expression. Localization of *Wolbachia* proteins was monitored in live yeast at 6 h and 24 h postinduction by live observation on a Nikon E800 fluorescence microscope with a 40× oil objective and processed using Metamorph imaging software (Molecular Devices). To determine colocalization of the GFP fusion protein with either actin or nuclei, yeast were fixed in either 4% paraformaldehyde or Karnovsky fixative for 20 min at room temperature after a 6-h induction and imaged using a 60× objective. Staining with rhodamine-labeled phalloidin (Invitrogen) to visualize the actin cytoskeleton was performed as previously described (78), and staining with 4′,6-diamino-2-phenylindole (DAPI) in mounting medium (SlowFade Gold; Invitrogen) allowed for visualization of nuclei.

### Yeast protein expression and Western blot assays.

Yeast harboring expression vectors containing proteins of interest were grown overnight in selective synthetic medium containing 2% glucose. These cultures were diluted to an OD_600_ of 1.0 in synthetic medium containing 4% galactose for 6, 16, or 24 h before cells were harvested by centrifugation and frozen at −80°C. Frozen yeast pellets were disrupted using bead beating (lysing matrix C on an MP FastPrep system; 20 s at speed 6) in 750 µl of lysis buffer (150 mM NaCl, 1% Triton X-100, 50 mM Tris-HCl [pH 8]) supplemented with HALT protease inhibitor cocktail and 5 mM EDTA (Thermo Scientific). Lysates were centrifuged at 10,000 × *g* for 1 min at 4°C to pellet cell debris, and supernatants were used for subsequent Western blot assays.

Proteins were separated on 4-to-20% Tris-glycine NB precast gels (NuSep) and transferred to a polyvinylidene difluoride membrane in Tris-glycine transfer buffer with 15% methanol at 40 V on ice for 3 to 4 h. The membrane was blocked for 5 min in starting block T20 blocking buffer (Thermo Scientific), followed by incubation with antibody (for 1 h at room temperature or overnight [O/N] at 4°C) according to standard protocols. SuperSignal West Pico chemiluminescent substrate was used to detect horseradish peroxidase (HRP) on immunoblots. Blots were reprobed after stripping in 100 mM glycine, 0.15 ND-40, 1% SDS, pH 2, for 1 h at room temperature and then O/N at 4°C. A PageRuler prestained protein ladder (Thermo Scientific) was used as a molecular mass marker. Antibodies utilized included anti-actin at 1:1,000 (LMAB-C4; Seven Hills BioReagents), anti-GFP–HRP conjugate at 1:5,000 (Miltenyi Biotec), and anti-phosphoglycerate kinase at 1:10,000 (Invitrogen).

### Actin sedimentation and bundling assays.

WD0830 was heterologously expressed in *Escherichia coli* (GenScript) (see Fig. S1 in the supplemental material) and centrifuged at high speed (150,000 × *g*) for 1 h at 4°C before use. The supernatant was then used in actin sedimentation assays with purified rabbit skeletal actin (Cytoskeleton, Inc.). Actin was stored in G buffer before use (5 mM Tris-HCl [pH 8.0], 0.2 mM CaCl_2_, 0.2 mM ATP, and 0.5 mM dithiothreitol). Polymerization was induced by the addition of 50 mM KCl, 2 mM MgCl_2_, and 1 mM ATP (final concentrations). The total amount of actin used in each assay mixture was kept constant (40 µl of a 1-mg/ml stock added to each reaction mixture). Either WD0830 (at a 40-ng/ml final concentration), the actin binding protein alpha-actinin (Cytoskeleton, Inc.; used as a positive control for F-actin binding and sedimentation), BSA, or nothing additional (negative controls) was added to individual actin samples. These mixtures were first centrifuged at 14,000 × *g* for 1 h at 24°C (to identify actin-bundling activity) and then centrifuged at 150,000 × *g* for 1.5 h at 24°C (to identify actin binding). Laemmli buffer was added to the supernatants, and pellets resulting from this centrifugation and these samples were run on an SDS-PAGE gel to visualize the proteins via silver stain. The gel lanes were scanned, and densitometry was measured using ImageJ software. To image actin filaments, F-actin was prepared as described above and, before centrifugation, stained with Acti-stain 555 fluorescent phalloidin (Cytoskeleton Inc.).

### *Drosophila* immunohistochemistry and microscopy.

Ovaries for immunolocalization were dissected in phosphate-buffered saline (PBS) solution 4 days after fly eclosion. We used published protocols for fluorescence *in situ* hybridization (22), with the following modifications: postfixation in 4% paraformaldehyde in diethyl pyrocarbonate (DEPC)-treated PBS, and ovaries were dehydrated in methanol and stored overnight at −20°C. In the morning, washes in DEPC–PBS-Tween (PBST) preceded a 5-min proteinase K treatment (0.05 mg/ml) at 37°C before incubation in hybridization buffer (50% formamide, 5× SSC [1× SSC is 0.15 M NaCl plus 0.015 M sodium citrate], 250 mg/liter salmon sperm DNA, 0.5× Denhardt’s solution, 20 mM Tris-HCl, and 0.1% SDS). Universal bacterial probe EUB338 conjugated to Alexa488 (Molecular Probes) was used to detect *Wolbachia* in the ovarioles. For F-actin detection we used rhodamine-labeled phalloidin or Acti-stain 488 fluorescent phalloidin (Cytoskeleton, Inc.), depending on the cross and the wavelengths utilized. Hybridized ovaries were mounted in Slow Fade Gold plus DAPI antifade reagent (Invitrogen).

Images were taken as *z*-series stacks at 1.5-µm intervals using a Nikon E800 fluorescence microscope with a 40× oil objective and processed using Metamorph imaging software (Molecular Devices). Care was taken such that exposure times were normalized across all experiments. For quantification of both *Wolbachia* within the developing oocyte and actin ring canal staining intensity, maximum projections from stacks generated were used, and we excluded the peritoneal sheath. The irregular blob tool was used to outline the entire oocyte, using DAPI staining as a guide. For quantification of actin ring canal intensity, the oval tool was used to outline ring canals adjacent to the developing oocyte.

### Transgenic *Drosophila* stocks and staging of flies.

Codon-optimized WD0830 constructs were generated using the Gateway pENTR-D/TOPO system (Invitrogen) as described in the user manual and transformed into One Shot Top10 competent cells (Invitrogen) using standard protocols. Correct entry vectors were used in combination with the pPRW destination vector (obtained from T. Murphy, *Drosophila* Genomics Resource Center; plasmid stock 1137; the vector features a Gateway cassette, UASp promoter, N-terminal monomeric RFP [mRFP], and mini-white [complement]) in an LR clonase reaction mixture [Invitrogen] as described in the user manual), and these resultant expression vectors were verified by restriction enzyme digests and sequencing. These constructs result in an N-terminal mRFP tag for WD0830. The purified plasmids were injected into *Drosophila* embryos (BestGene, Inc.). Thirteen independent lines on the X, second, and third chromosomes were recovered. Standard methods were used for all crosses and culturing. The following stocks were obtained from the Bloomington *Drosophila* Stock Center (BDSC) at Indiana University (http://flystocks.bio.indiana.edu). Stock 145, which carries *W^1^*, was used as the *Wolbachia*-infected control line for characterization of WD0830 expression over the course of development. The three *Wolbachia*-containing Gal4 driver stocks from BDSC used were as follows: Oskar driver, w[1118]; P A11/CyO (BDSC 44241); Maternal Triple driver (MTD), P{w[+mC]=otu-GAL4::VP16.R}1, w[*]; P{w[+mC]=GAL4-nos.NGT}40; P{w[+mC]=GAL4::VP16-nos.UTR}CG6325[MVD1] (BDSC 31777); and Maternal Alpha-Tubulin 67C driver, w[*];P{w[+mC]=matα4-GAL-VP16}V37 (BDSC 7063).

Of the thirteen insertion stocks carrying pPRW-WD0830 in a *w*[1118], *Wolbachia*-positive background and named P{w[+mC]=UASp-RFP.WalE1} (BestGene, Inc., Chino Hills, CA, USA), homozygous viable insertions P{w[+mC]=UASp-RFP.WalE1}2M (chromosome 2), -4M (chromosome 3), -6M (chromosome 3), and -7M (chromosome 3) were examined most extensively. Oskar-GAL4 driver and P{w[+mC]=UASp-RFP.WalE1}6M stocks were crossed for quantification of actin, *Wolbachia*, and localization of RFP-WD0830. *Wolbachia* infection status for stocks acquired from the BDSC and from BestGene, Inc., was determined via PCR.

### Nucleic acid extraction and quantitative PCR.

To identify *Wolbachia* titers within embryos from mothers expressing *walE1*, individual embryos were homogenized in 10 µl of water, and this lysate was diluted 1:100 for quantitative PCR. Additionally, pools of 20 to 30 embryos were subjected to DNA extraction (using the Qiagen DNeasy blood and tissue kit), and nucleic acids were diluted to <20 ng total for qPCR. Quantitative PCR was performed on this DNA to determine the *Wolbachia* titer (with reference to the host) using an Applied Biosystems StepOne real-time PCR system and Sybr green chemistry (Applied Biosystems). We used *w*sp primers for *Wolbachia* (forward, CATTGGTGTTGGTGTTGGTG; reverse, ACCGAAATAACGAGCTCCAG) and Rpl32 primers for the host (forward, CCGCTTCAAGGGACAGTATC; reverse, CAATCTCCTTGCGCTTCTTG) with the following cycling conditions: 95°C for 10 min, then 40 cycles of 95°C for 15 s and 60°C for 1 min. To characterize *walE1* expression throughout fly development, RNA and DNA were extracted from individual flies (stock 145) at different stages of development by using a modified TRIzol extraction protocol. Briefly, 500 µl of TRIzol was added to flies and samples were homogenized using a pestle. After a 5-min incubation at room temperature, a 12,000 relative centrifugal force centrifugation (at 4°C for 10 min) was followed by a chloroform extraction. The aqueous phase containing RNA was extracted a second time with phenol-chloroform before isopropanol precipitation of RNA. This RNA pellet was washed and resuspended in RNA storage solution (Ambion). DNA extraction from the same flies was performed using ethanol precipitation of the organic phase during the first chloroform extraction. To detect the number of *walE1* transcripts, we utilized the RNA extracted from these flies and the SensiFAST Syber Hi-ROX one-step RT mix (Bioline) and the Applied Biosystems StepOne real-time PCR system with the following primer set: WalE1F, TGGGAAGAAAAGGCTCTGAA; WalE1R, TCAATGAGGCGCTTCTAGGT. As a reference for transcription activity of the core *Wolbachia* genome, we utilized the *Wolbachia ftsZ* gene (forward, TTTTGTTGTCGCAAATACCG; reverse, CCATTCCTGCTGTGATGAAA). We did not employ the *w*sp qPCR primer sets, as *w*sp’s function is unclear and we do not know if *w*sp is stably expressed during development or in different tissues. We therefore designed primers to FtsZ, because as a core protein involved in cell division, the quantities of FtsZ would better correlate with bacterial numbers and activity. Reactions were performed in duplicate or triplicate in a 96-well plate, and calibration standards were used to calculate primer efficiencies. These efficiencies, along with the cycle threshold values generated by the machine, were used to calculate the relative amounts of *Wolbachia*, by using the ΔΔ*C_T_* (Livak) and Pfaffl methods (79).

## SUPPLEMENTAL MATERIAL

Figure S1  Heterologously expressed GFP-WD0830 was detected in yeast via Western blotting (anti-GFP antibody). Yeast were induced to express the protein by incubation in galactose (2%)-containing medium for 24 h. Download Figure S1, DOCX file, 1 MB

Figure S2  His-tagged WD0830 was heterologously expressed in *E. coli* and purified (GenScript). (A) Lane 1, BSA control; lane 2, WD0830. (B) Lane 3, anti-His Western blot results for His-WD0830. Download Figure S2, DOCX file, 0.2 MB
