# Supplementary material for: Identification and Characterization of a Candidate Wolbachia pipientis Type IV Effector That Interacts with the Actin Cytoskeleton
Source: mBio. 2016 Jul 5;7(4):e00622-16. doi: 10.1128/mBio.00622-16 (PMC4958246; doi:10.1128/mBio.00622-16)
Supplement: Figure S1 — Heterologously expressed GFP-WD0830 was detected in yeast via Western blotting (anti-GFP antibody). Yeast were induced to express the protein by incubation in galactose (2%)-containing medium for 24 h. Download [file mbo003162882sf1.docx]

**Supplementary Figure 1.** Heterologously expressed GFP-WD0830 is detected in yeast by Western blot (α-GFP). Yeast were induced to express the protein by incubation in galactose (2%) containing media for 24 hours.
