# Supplementary material for: Identification and Characterization of a Candidate Wolbachia pipientis Type IV Effector That Interacts with the Actin Cytoskeleton
Source: mBio. 2016 Jul 5;7(4):e00622-16. doi: 10.1128/mBio.00622-16 (PMC4958246; doi:10.1128/mBio.00622-16)
Supplement: Figure S2 — His-tagged WD0830 was heterologously expressed in E. coli and purified (GenScript). (A) Lane 1, BSA control; lane 2, WD0830. (B) Lane 3, anti-His Western blot results for His-WD0830. Download [file mbo003162882sf2.docx]

**Supplementary Figure 2.** His tagged WD0830 was heterologously expressed in *E. coli* and purified (Genscript). (A) lane 1 – BSA control, lane 2, WD0830. (B) Lane 3 = anti-his western blot of his-WD0830.
